# A Morning Star

**Published:** 1895-06

**Authors:** 


### EDITORIAL.

### A Morning Star.

Recently a beautiful "Morning Star" came to the Editor of the DENTAL REGISTER, borne in the uplifted right hand of a beautiful Bronze Statue, which had been fashioned by most exquisite Art.

Where did it come from?

It came from the warm, loving hearts of our Confreres of the Odontological Society of Cincinnati.

Upon the Pedestal of the Statue there is appended a silver Tablet bearing the following inscription:

"PRESENTED TO

DR. JONATHAN TAFT

BY THE

ODONTOLOGICAL SOCIETY OF CINCINNATI.

In appreciation of his Efforts to Elevate the

Profession of Dentistry during a period of Fifty Years.

April 30th 1895."

This Figure, beautiful, exquisite and valuable as it is, is as nothing, to the recipient, compared with the Spirit and Regard that prompted the presentation.

The Memory of the occasion, and that which it represents can never be effaced while life lasts.

My Friends, May it be done to you as you have done to another; and may your cup of happiness be as full as his has been made, by this act of yours.

